# Quack Medical Literature in Religious Family Newspapers

**Published:** 1864-04

**Authors:** 


### QUACK MEDICAL LITERATURE IN RELIGIOUS FAMILY NEWSPAPERS.

We have heretofore entered our protest against the iniquity of a large portion of the religious press of the day, in advertising quack nostrums. Without further comment at present, we quote a very appropriate and truthful article, which we find in the *Round Table*, of a recent date:

"A Short Word with the Religious Press.—It is not a matter of especial wonder when a traveler writes that he saw emblazoned, in huge letters, upon some of the old ruins of Greece, the advertising cards of quack medicines. As Americans, we are pretty thoroughly educated to a point of resignation and indifference, when we find huge bulletins despoiling monuments of art and beauty, and even when they stare us in the face on rocks and hillsides during our summer tours of respite and recreation. Nor does it disturb the exquisite, as it once did, to be obliged to read a daily mixture of criminal news and the disgusting advertisements of the medicine venders. All this we are becoming inured to as a people. But there is one medium of publicity where we look for something higher, purer, better. There is one source of power whence we look to see only healthful streams departing. If the religious press of the country fails to stem the tide, how can we hope to see any effort at restraint in other quarters? If the Christian editors and publishers of the land are false to their high calling and duty, what shall prevent the lifting up of the flood-gates, and the outpouring of a deluge of filth and pollution?

The facts of the case are apparent to every pure-minded man who reads the weekly religious press. Before us are recent issues of two leading religious journals, the *Independent* and the *Observer*. We find in each broad columns staring us in the face, full-freighted with the disgusting details of the properties of certain medicines. "Helmbold's Buchu," "Constitution Water," and "Cherokee Injections," are instances of the most revolting. And these are spread out through long columns, and sent forth under the name and with the sanction and influence of the religious press. They go into the best families of the land, to be read in the pure atmosphere of the family circle and about peaceful and wholesome Christian firesides. They carry disgust to the modest, and tend to aggravate and increase vice and crime.

We protest against these growing indecencies of our religious journalism. And in doing this, it is but simple justice to say that all the weekly religious papers do not thus prostitute their columns. There are several worthy exceptions. But it is a matter of regret that any journals which have attained to a great circulation and influence should go forth from week to week, professedly the religious expounders of the hour, but practically mere money-sheets, laden with purchased puffs and shameless advertisements. Perhaps if less attention was paid to financial successes and more to the possible good to be done in the way of a stronger and healthier Christian literature, they might find quite as many friends, and surely more nearly accomplish the supposed object of their existence.

This we say with a heart in sympathy with every effort that may tend to make men better, purer, happier. We say it not merely inspired by disgust at the presentation of such indecent advertisements at our own counter, making us doubly ashamed when assured that certain religious papers made no objection to their publication, but rather actuated by a desire to see these great mediums of power and influence working from a higher motive than mere money success, and looking to a grander end to be accomplished than the pleasing and tickling and puffing of men. Christianity can need no help bought with the profits of such indecency. The cause of humanity demands a literature which shall inspire a truer, purer life.—*Cincinnati Lancet and Observer.*

